# ABIDA: An Automated Brain Imaging Data Processing and Analysis Platform

**DOI:** 10.1111/cns.70552

**Published:** 2025-08-01

**Authors:** Han Yang, Min‐Jie Zhang, Pu‐Xin Sun, Xi‐Jian Dai

**Affiliations:** ^1^ Department of Radiology The Second Affiliated Hospital, Jiangxi Medical College, Nanchang University Nanchang China; ^2^ Jiangxi Provincial Key Laboratory of Intelligent Medical Imaging Nanchang China

## Abstract

Addressing RS‐fMRI toolkit limitations—notably operational complexity and expertise barriers—this study introduces ABIDA toolbox. Integrating cross‐toolkit functions with intelligent algorithms and quantitative metrics enhances workflow through intuitive design and automation.

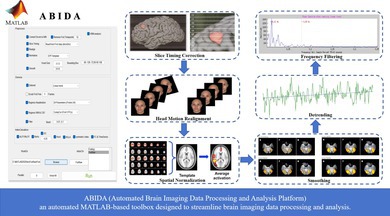

1

Resting‐state functional magnetic resonance imaging (RS‐fMRI) is a method to study the spontaneous neural activity in the brain in the resting state, which can reflect the pathophysiological changes of the basic brain function and is especially suitable for clinical research. In recent years, with the progress of imaging data processing methodology, the imaging studies of resting brain function have made a breakthrough, which can be used to explore not only the basic physiological functional networks of the human brain but also the changes of the structural networks related to spontaneous brain neural activity in diseases. A number of comprehensive software packages have been developed for RS‐fMRI data analysis based on MATLAB, such as RS‐fMRI data analysis Toolkit (REST [1]), RESTplus [2] and DPABI [3]. However, REST has not been updated for a long time. RESTplus and DPABI require complex parameter settings and basic knowledge of data analysis. The complexity of software operations hinders the efficiency and standardization of data processing, especially for the medical community who lacks a programming base. In response to this need, we have developed a new user‐friendly toolbox (Figure 1), named automated brain imaging data processing and analysis platform (ABIDA), which is based on the MATLAB language, integrates the basic functions of REST, DPARSF, and RESTplus, and adds more intelligent algorithms and RS‐fMRI metrics. The toolkit, an exemplar dataset, and manual were shared. The ABIDA greatly simplifies the data analysis process, significantly improves the efficiency of data preprocessing, and standardizes the data analysis process. In comparison to existing software of an analogous nature, ABIDA exhibits the following notable advantages:

1. ABIDA employs a straightforward and user‐friendly interface, which streamlines the numerous intricate data processing steps into straightforward and accessible options, enabling users to expeditiously complete data input, pre‐processing, and analysis through a simple interface, which significantly improves the user experience.
2. Flexible pipelined preprocessing steps. These preprocessing steps contain all mainstream processing steps, including data format conversion, time point removal, slice timing correction, head realignment (motion and rotate), spatial normalization, smoothing, de‐trending, bandpass harmful covariate regression, and filtering.
3. New RS‐fMRI metrics calculation. The toolkit can calculate not only commonly used RS‐fMRI metrics such as amplitude of low‐frequency fluctuation (ALFF) and regional homogeneity (ReHo) and structured data by voxel‐based morphometry (VBM) but also some new RS‐fMRI metrics, named Hurst, RSLA, and symmetric index.
4. The ABIDA program incorporates a highly automated pre‐processing procedure that automatically handles multiple steps, from data pre‐processing to postprocessing metrics calculation. If the input image data is in a Digital Imaging and Communications in Medicine (DICOM) format, the toolkit will automatically identify the parameters and perform the data analysis according to the optimal parameter settings, instead of manually setting parameters (Figure 1A). Users can also choose to set parameters manually, such as when they enter data in NIFTI format. Figure 1B shows the pre‐processing generated folders. Using the “FunRaw” file as the initial file as an example: (1) Convert the DICOM files to the NIFTI format to generate the “FunImg” file; (2) a file with a suffix of added a capital letters will be generated after each processing step‐ “A” represents slice timing, “R” represents head motion realign, “W” represents normalize, “S” represents smooth, “D” represents detrend, “C” represents WM/CSF signal regression, and “F” represents filter. This naming convention encoded in the form of words can intuitively track the preprocessing stage, thereby improving the transparency of the data source in the analysis workflow.

**FIGURE 1:**
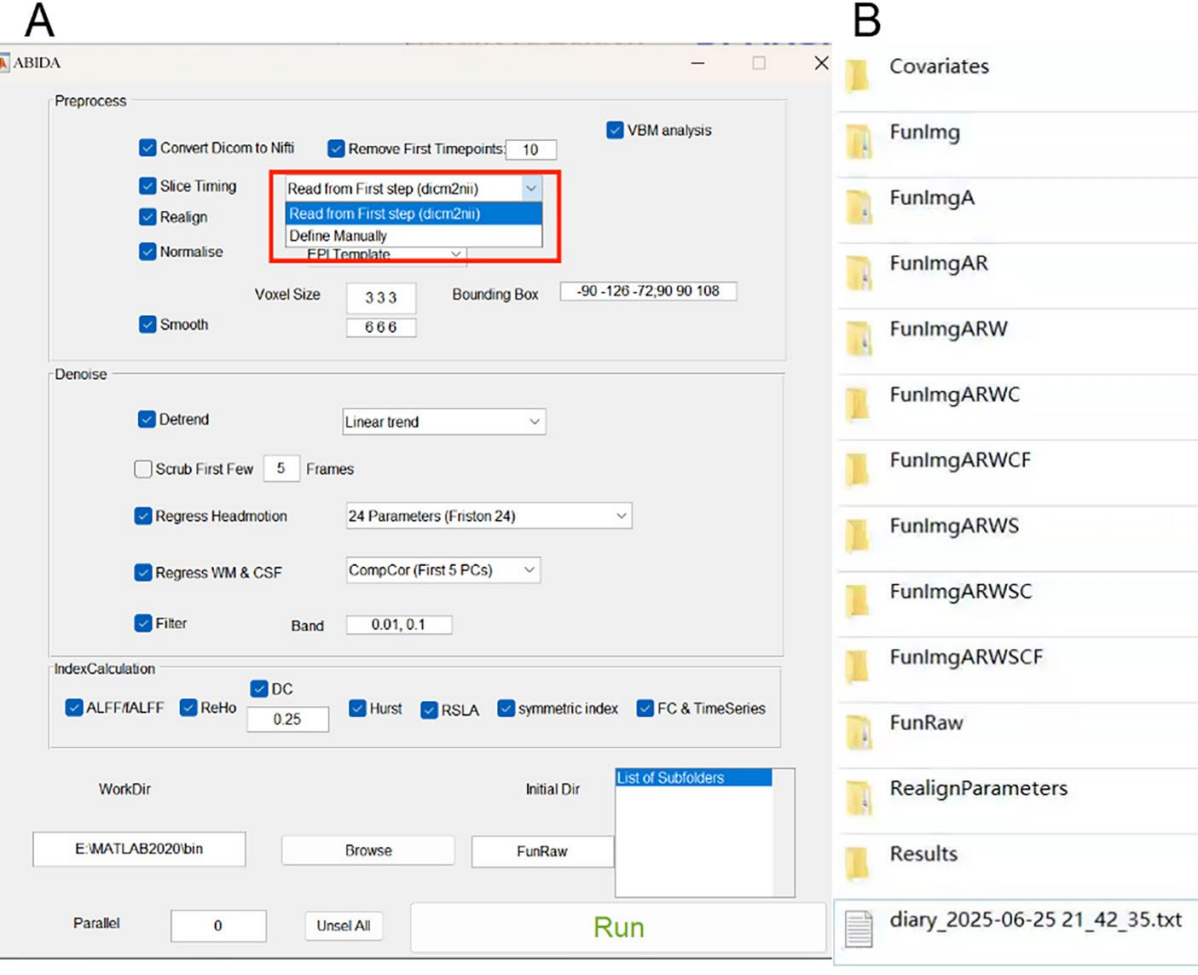
Toolbox GUI interface (A) and pre‐processing generated folder (B).

5 The toolkit can synchronously calculate all RS‐fMRI metrics (ALFF, ReHo, degree centrality, Hurst, RSLA, symmetric index and functional connectivity), and the smooth step will be performed where appropriate.
6 Traditional toolkits require that data be stored in a folder specifically named “FunRaw”, but our toolkit has abandoned this nomenclature restriction. However, the restriction of data organization structure are the same, including the folder hierarchy, file format specifications, or metadata annotation requirements.
7 In terms of computational performance and work efficiency, ABIDA also has significant advantages. The integration and automation of data processing greatly reduce unnecessary work delays, thereby significantly improving time efficiency.

In conclusion, ABIDA offers superior automated integration and optimized processing capabilities compared to traditional software packages, making it a more efficient, straightforward, and powerful toolbox.

## Disclosure

No artificial intelligence‐generated content (AIGC) tools were employed in the creation of any substantive material within this scholarly work.

## Conflicts of Interest

The authors declare no conflicts of interest.

## Data Availability

The authors have nothing to report.
